# Identification of recurrent genetic patterns from targeted sequencing panels with advanced data science: a case-study on sporadic and genetic neurodegenerative diseases

**DOI:** 10.1186/s12920-022-01173-4

**Published:** 2022-02-10

**Authors:** M. Tarozzi, A. Bartoletti-Stella, D. Dall’Olio, T. Matteuzzi, S. Baiardi, P. Parchi, G. Castellani, S. Capellari

**Affiliations:** 1 Department of Medical and Surgical Sciences, University of Bologna, Bologna, Italy; 2 Department of Experimental, Diagnostic and Specialty Medicine, University of Bologna, Bologna, Italy; 3 IRCCS Institute of Neurological Sciences of Bologna, Bologna, Italy; 4 Department of Physics and Astronomy, University of Bologna, Bologna, Italy; 5 Department of Biomedical and Neuromotor Sciences, University of Bologna, Bologna, Italy

**Keywords:** NGS, Genetic modifiers, Polygenic score, Gene panels, Machine learning, Complex diseases, Neurodegeneration, CJD, Alzheimer’s Disease

## Abstract

**Background:**

Targeted Next Generation Sequencing is a common and powerful approach used in both clinical and research settings. However, at present, a large fraction of the acquired genetic information is not used since pathogenicity cannot be assessed for most variants. Further complicating this scenario is the increasingly frequent description of a poli/oligogenic pattern of inheritance showing the contribution of multiple variants in increasing disease risk. We present an approach in which the entire genetic information provided by target sequencing is transformed into binary data on which we performed statistical, machine learning, and network analyses to extract all valuable information from the entire genetic profile. To test this approach and unbiasedly explore the presence of recurrent genetic patterns, we studied a cohort of 112 patients affected either by genetic Creutzfeldt–Jakob (CJD) disease caused by two mutations in the *PRNP* gene (p.E200K and p.V210I) with different penetrance or by sporadic Alzheimer disease (sAD).

**Results:**

Unsupervised methods can identify functionally relevant sources of variation in the data, like haplogroups and polymorphisms that do not follow Hardy–Weinberg equilibrium, such as the *NOTCH3* rs11670823 (c.3837 + 21 T > A). Supervised classifiers can recognize clinical phenotypes with high accuracy based on the mutational profile of patients. In addition, we found a similar alteration of allele frequencies compared the European population in sporadic patients and in V210I-CJD, a poorly penetrant *PRNP* mutation, and sAD, suggesting shared oligogenic patterns in different types of dementia. Pathway enrichment and protein–protein interaction network revealed different altered pathways between the two *PRNP* mutations.

**Conclusions:**

We propose this workflow as a possible approach to gain deeper insights into the genetic information derived from target sequencing, to identify recurrent genetic patterns and improve the understanding of complex diseases. This work could also represent a possible starting point of a predictive tool for personalized medicine and advanced diagnostic applications.

**Supplementary Information:**

The online version contains supplementary material available at 10.1186/s12920-022-01173-4.

## Background

Gene panels are a powerful clinical and research tool that allow to perform massively parallel sequencing on a set of genes of interest. This technology is often used in the clinic practice as a diagnostic tool, however, at present a large fraction of the collected genetic information remains unexploited, since their valence is difficult to assess. On the research side however, genetic modifiers and oligogenic patterns of inheritance are gaining an increasing interest, because of the phenotypic variability of some diseases and as a possible answer to missing heritability of other conditions [1]. An increasing number of papers is pointing out that the genetic part of the missing information about heritability and phenotypic heterogeneity is likely to be addressed by a set of variants reinforcing themselves in connected molecular pathways rather than a specific mutation yet to be discovered [1–3]. It is therefore of great relevance to improve methods of analysis to acquire a richer insight of the overall genetic features. For this reason, in the genomic field there is an increasing use of machine learning methods (ML) and network analysis, which allow to identify recurrent genetic patterns in the data and to integrate and amplify single genetic variants in their biological context [4–10]. Neurodegenerative brain diseases are progressive and fatal conditions primarily affecting the central nervous system. In the last decades, linkage studies in families with a disease showing Mendelian inheritance identified high-penetrant mutations in causal genes in a minority of them. In the vast majority, common variants in genes with significant associations in genome-wide association studies (GWAS) concurred, with a modest increase in disease risk, to the disease. Genetic risk factors or modifiers play an important role both as additional risk factors in co-occurrence with incompletely penetrant mutations but also as modulators of disease severity, age of onset and in the overall course of the disease [11–15]. Here, we consider three inheritance models in two neurodegenerative diseases, genetic Creutzfeldt–Jakob Disease (gCJD) and sporadic Alzheimer Disease (sAD). Creutzfeldt–Jakob Disease (CJD) is the most common human prion disease [16], where genetic forms caused by mutations in the *PRNP* gene account for 15% of the cases and show autosomal dominant inheritance with variable penetrance [17]. We focused on the two most common *PRNP* mutations in the Italian population, the highly penetrant p.Glu200Lys (E200K group) and the p.Val210Ile (V210I group), that shows low penetrance [18]. The *PRNP* gene, located in chromosome 20 in the human genome, is 16 Kb long and made up of two exons, the second containing the whole open reading frame, resulting in a mature protein of 208 amino acids. The most important known risk factor and phenotypic modifier is the polymorphism at the codon 129 of the *PRNP* gene, that can result either in Methionine or Valine. Homozygotes are overrepresented in the population affected by prion diseases, while heterozygosity has a protective role. Sporadic Alzheimer Disease is the third model considered in this work: sAD is known to be influenced by both genetic and environmental factors. Extensive studies led to the discovery of important predisposing factors, such as *APOE* genotype, and variants in *ABCA7, SORL1, TREM2* genes [19]; nevertheless the missing heritability of sAD remains an important open question [20, 21]. In this study we tried to improve current approaches towards target sequencing data analysis considering each single nucleotide variant (SNVs) and small indels obtained through DNA target sequencing of twenty-nine genes known to play a role as risk factors or determinants of dementias, on one-hundred and twelve patients affected by either sAD or gCJD caused by either the highly penetrant mutation p.Glu200Lys or a lowly penetrant p.Val210Ile mutation. This study employs a data analysis workflow involving a combination of statistical, machine learning and network analysis to extract all the valuable information to identify and evaluate potential polygenic contributions to neurodegenerative dementia. We focused on differences of recurrent genetic patterns covered by our gene panel between groups of interest, sAD vs gCJD, and in the CJD group between the two described mutations, p.Glu200Lys and p.Val210Ile. As the results of our case study show, this workflow represents a suitable approach to acquire a deeper understanding of the genetic pattern present in target sequencing data, able to improve our understanding of the underlying molecular biology of complex diseases and a possible starting point as a predictive tool for personalized medicine applications.

## Materials and methods

### Subjects

We recruited patients with definite, probable, probable laboratory-supported, and possible CJD or AD diagnosed according to National Institute of Aging/Alzheimer's Association (NIA/AA) [22] or International Working Group-2 (IWG-2) [23] for AD and updated clinical diagnostic criteria for sporadic Creutzfeldt–Jakob disease [24], afferent to the Cognitive Disorders and Dementia Center of the UOC Clinica Neurologica, Bologna, either as outpatients, inpatients, or sent for genetic analysis between 2010 and 2019. One-hundred-twelve patients with either gCJD (n = 66) or sAD (n = 46) were recruited. Among the sixty-six gCJD patients, forty were carriers of the p.Val210Ile and twenty-six of the p.Glu200Lys mutation. For brevity, in this work we will refer to these groups of patients as V210I and E200K groups, whilst specific protein coding variants will be reported with the aminoacidic shift nomenclature and non-coding variants with nucleotide shift nomenclature. Ethical approval was obtained from the ethical board of our institution. For all subjects, written informed consent was provided. All methods were performed in accordance with the relevant guidelines and regulations.

### DNA extraction

Genomic DNA from peripheral blood was extracted using the Maxwell 16 extractor (Promega, Madison, WI, USA) and quantified using the Quantus Fluorometer (Promega) with QuantiFluor double-stranded DNA system.

### Target sequencing and secondary analysis

Target sequencing covers 29 genes (Additional file 1: Table S1 see also Bartoletti-Stella et al. 2018 [25]), known to play a role as risk factors or primary determinants in different types of dementia [26]*.* Libraries were constructed with the amplicon-based assay TruSeq Custom Amplicon v1.5 (TSCA, Illumina, CA, USA), sequencing was performed on a MiSeq sequencer using Illumina V2 reagent kit, using 2 × 150 bp paired end read cycles. Raw data were analyzed by the MiSeq Reporter software (Illumina), aligned to GRCh37/Hg19 using bwa-mem with variant calling and depth of coverage calculation with Genome Analysis Toolkit (GATK) [27]. During the variant calling steps, variants were filtered based on quality using Q30 as threshold, which means that at most 0.1% error rate is allowed.

### Data transformation

To obtain an input suitable for the computational and statistical analysis, containing the whole genetic variability in the dataset and still maintaining the single-patient detail, the genetic information contained in the Variant Call Format (VCF) files was transformed into binary data through an in-house Python script. Our script generates a matrix in which each row represents a variant reported in the provided VCF files at least once and each column is named after an ID assigned to each patient. In the matrix, 0 indicates that the variant is not present in the VCF file of the patient whereas 1 indicates its presence. A second version of the script was also used to produce a ternary matrix in which the zygosity information was added, thus 1 indicates heterozygosity and 2 homozigosity. On these matrices, machine learning methods and statistical analysis were applied using scikit-learn [28], seaborn [29] and plotly express [30] packages on Jupyter notebooks.

### Machine learning analysis

We used both supervised and unsupervised methods to extract as much valuable information as possible from our transformed data. To visualize such high dimensional data, we tested different dimensionality reduction techniques, such as Principal component analysis (PCA), t-distributed stochastic neighbour embedding (t-SNE) using Jaccard similarity as metric. As supervised methods, we used decision trees on binary data labelled accordingly to the disease or genotype (sporadic, p.Glu200Lys, p.Val210Ile) of each patient. The classifier was trained on a random selection of 2/3 of the dataset and adequate branching depth was set to avoid overfitting. The classification rules were tested on a validation set represented by the remaining 1/3 of the dataset.

### Statistical analysis

Allele frequencies of each variant found at least in one patient of our cohort were obtained by the ternary matrix in which the zygosity information was included. We then compared allele frequencies calculated in the sAD and in the gCJD populations with those reported in the gnomAD database [31] for the non-Finnish European population using Fisher’s exact test and Benjamini–Hochberg multiple test correction. We defined *MAPT* haplotypes in our population using the two coding SNV rs1052553 and rs1800547 [32, 33], and we tested for Hardy–Weinberg equilibrium using the R package “HardyWeinberg” [34].

### Protein interaction network and pathway analysis

To further explore the biological interplay of genes harboring significant variants we performed protein–protein interaction network and pathway enrichment analysis. Protein–protein interaction network (PPIn) was built by merging data from four state-of-the-art protein–protein interaction databases [35–38]. All of them were obtained with high-throughput assays and comprise only biophysical interactions (i.e., molecular docking) between proteins [39]. The network resulting from their union covers almost 14,000 genes and 110,000 interactions between gene products. For each disease group, we built a group-specific subnetwork by mapping on the PPIn those genes harboring at least one variant with a p-adj < 0.05 and considering their nearest neighbor genes. We then focused on differences between group-specific subnetworks by identifying, for the group pairs of interest (i.e., sAD-gCJD, V210I-E200K), the sets of genes unique to one disease with respect to the other. On this sets of genes, we performed pathway enrichment using Gene Ontology [40, 41], to explore which pathways are likely to be affected by differences in genes harboring at least one statistically significant (p < 0.05) variant between disease groups.

## Results

### Unsupervised methods identify functionally relevant genetic modules

**Fig. 1:**
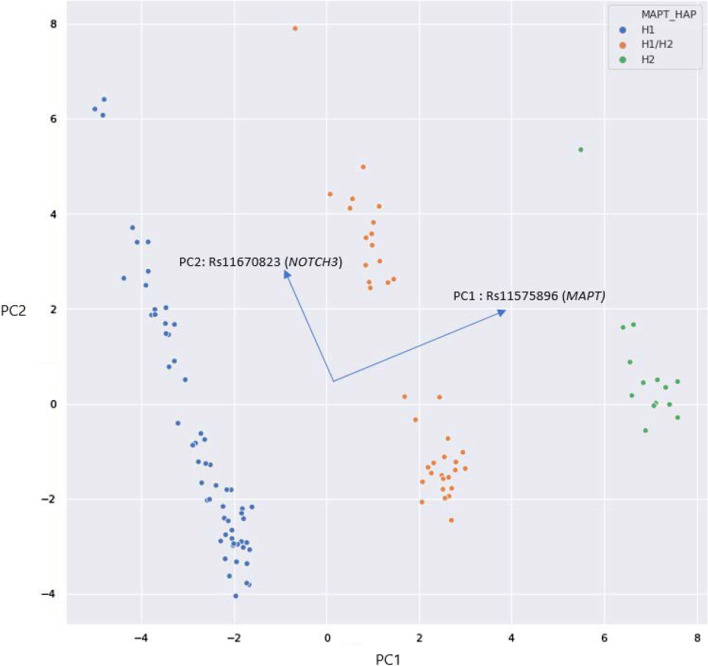
2D plot of the Principal Component Analysis (PCA) computed on the 1046 × 112 ternary matrix. PCA is a dimensionality reduction technique that computes an orthogonal linear transformation of the data to a new 2D coordinate system so that the greatest variance is on the x-axis (PC1) and the second greatest variance on y-axis. Each dot represents a patient, that is plotted in the 2D space accordingly to its genetic profile expressed in the ternary matrix. PC1 and PC2 show the main sources of variance in our data, accounting for 22% of overall variance, that are represented by variants on *MAPT* and *NOTCH3* genes, respectively. PCA plot and hierarchical clustering recognize clusters that correspond to the *MAPT* haplotypes on the x-axis, as shown by coloured labels in the picture legend. Similarly, the distribution along the y-axis matches haplotypes in the *notch3* gene (not shown)

Binary transformation of the genetic information contained in VCF files led to the generation of a matrix with shape 1046X112 where each row identifies a variant, and each column identifies a patient*.* The first exploratory analysis of the 1046X112 matrix containing the whole genetic information was performed trough dimension reduction with Principal Component Analysis (PCA) (Fig. 1). In the 2D plot each dot represents a patient. The first two principal components (PC1 and PC2) explain 22% of the overall variance of the dataset (Additional file 1: Table S3). PC1’s main contributors are a group of SNPs that are all harboured in the *MAPT* genomic region (Additional file 1: Table S2), that previous works have defined as haplotype-specific [33, 42]. The second principal component involves a more heterogeneous group of SNPs in which the SNP rs11670823 in the *NOTCH3* gene is the major contributor (Additional file 1: Table S3).

We labelled each sample according to the disease affecting the patient, the genotype (sporadic*,* p.Glu200Lys, p.Val210Ile*)* and a label marking a possible batch effect due to different sequencing runs. None of these labels matched the identified clusters (Additional file 1: Fig. S1). Based on the loadings and score values of the PCA we focused on the main genetic sources of variation in the dataset. We identified the two main *MAPT* haplotypes (H1,H2 and H1/H2), according to two coding SNPs rs1052553 and rs1800547 [33, 42] which are in linkage disequilibrium (LD) with the rs11575896 (first contributor to PC1, Additional file 1: Table S2). The result of the labelling of our dataset according to *MAPT* haplotypes perfectly matches the clusters in the PCA plot, as shown in Fig. 1. Our population is in Hardy–Weinberg (HW) equilibrium for the tested SNPs. The distribution on the y-axis recognizes specific SNPs patterns associated to haplotypes of *NOTCH3* (Additional file 1: Fig. S2). [43] Interestingly, the SNP rs11670823 (c.3837 + 21 T > A), which is the major contributor to the PC2, is in LD with three *NOTCH3* haplotype defining SNPs (rs1044009, rs104423702 and rs4809030) [43], and is in HW disequilibrium (p = 0.03) in the complete cohort (sAD p = 0.085, E200K p = 0.276, V210I p = 0.420).

### Supervised methods recognize clinical phenotypes with high accuracy

**Fig. 2:**
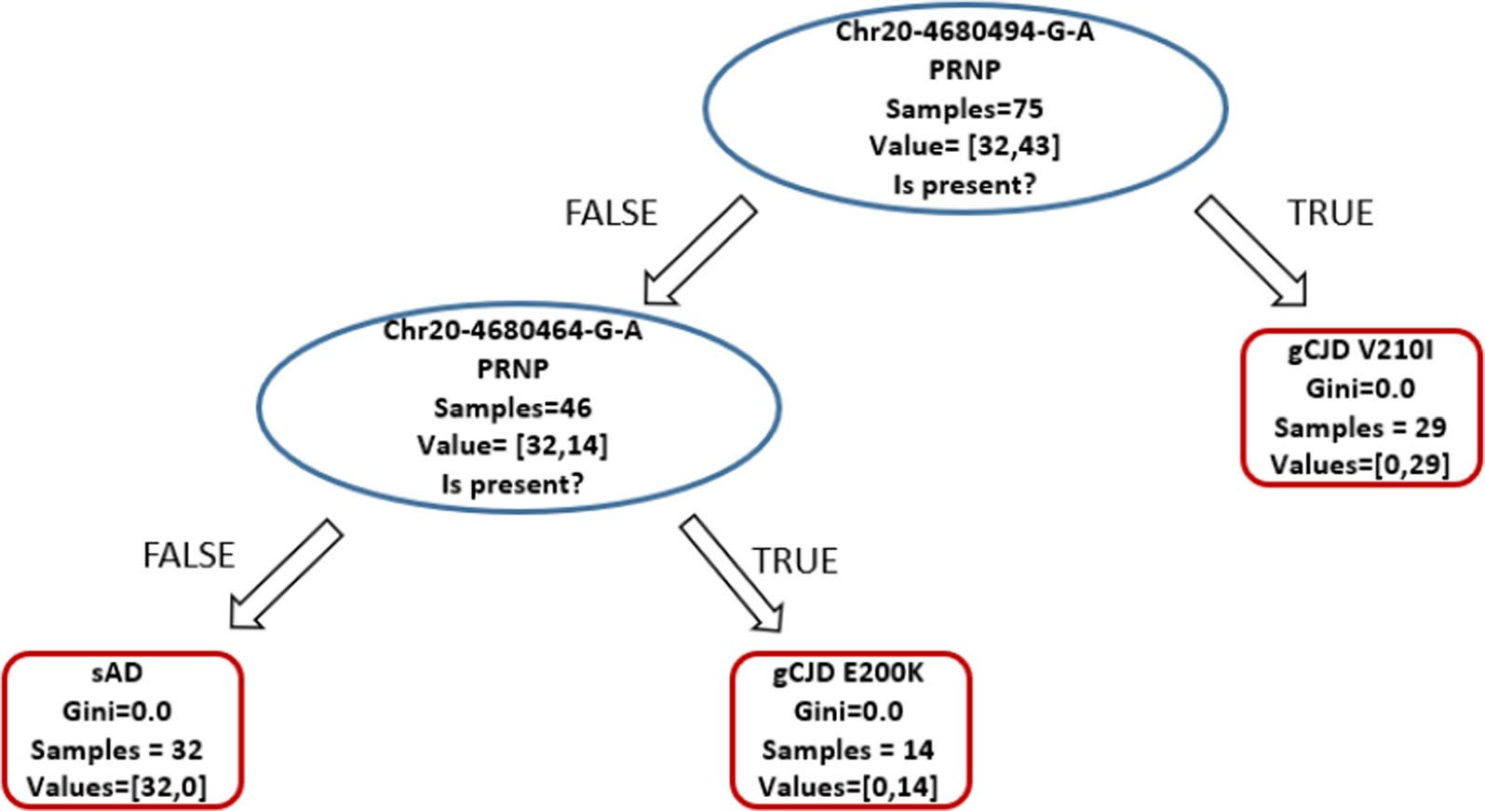
Dataset classification according to decision trees analysis: this supervised method computes on the 1046 × 112 matrix a classification based on the labels provided. The classifier correctly identifies the two disease groups on the two disease-causing mutations

Supervised classifiers were used for automatic recognition of genetic patterns among the 1046 variants identified in this dataset. In the 112X1046 matrix, to each sample a label corresponding to the disease (class: “CJD” or “AD”) was added. The classification was achieved perfectly, with 100% of accuracy (ratio of correctly predicted observation to the total observations) on the test set, basing the classification on the two disease-causing mutations p.Val210Ile and p.Glu200Lys (Fig. 2).

To test for the presence of additional recurrent genetic patterns that could characterize a homogeneously phenotypic group and possibly act as modifier, we removed from the input data provided to the classifier only the two rows of the 1046-rows matrix indicating the disease-causing mutations. As expected, accuracy decreased both in training set and in test set, but interestingly the classifier managed to distinguish the two diseases with a good accuracy (training = 0.97, test 0.78) (Table 1).

**Table 1:** Classification metrics.

	Precision	Recall	F1	Support
sAD	0.71	0.8	0.71	15
gCID	0.85	0.77	0.85	22

Precision is the ratio of correctly predicted observation to the total predicted positive observations (TruePositive/TruePositive + FalsePositive), Recall is the ratio of correctly predicted positive observations to all observations in actual class (TruePositive/TruePositive + FalseNegative), F1 Score is the harmonic mean of Precision and Recall (F1 Score = 2*(Recall * Precision) / (Recall + Precision)). Support indicates class numerosity

**Fig. 3:**
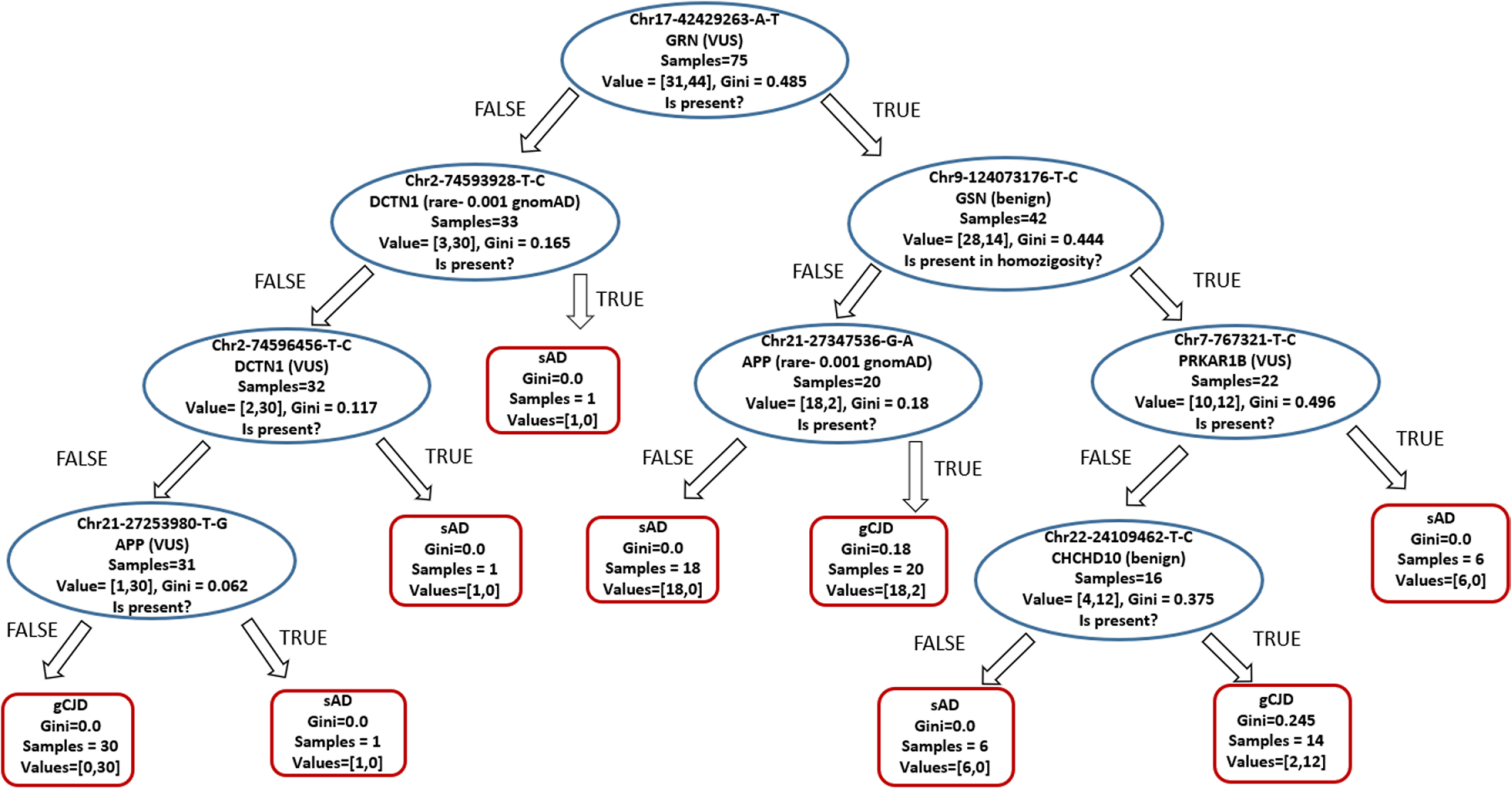
Result of Decision Trees analysis on the dataset deprived of the information about gCJD-causing mutations. Classification is accomplished with 0.71 accuracy for sAD and 0.85 for gCJD. Classification is based on the reported eight variants harboured in six genes. Four of these are variants of uncertain significance not reported in the GnomAD database harbored in the genes *APP* c.*1A > C (rs748508166), GRN c.1179 + 100A > T, *DCTN1* p.Lys519Glu, *PRKAR1B* c.595 + 369 T > C (rs1342588350), two of them are rare (Minor Allele Frequency < 0.05) variants in the European population, *APP* p.Phe435 = (rs148180403, MAF = 0.001), *DCTN1* p.Ala816 = (rs1130484, MAF = 0.007) and two are common benign variants in *CHCHD10* (c.261 + 99A > G) and *GSN* (c.666 + 53 T > C). “Value” indicates the number of samples at the given node that fall into each category. The “Gini” score quantifies the purity of the node/leaf, when greater than zero implies that samples contained within that node belong to different classes while a gini score of zero means that within that node only a single class of samples exist

The classification is based on eight variants involving six different genes (Fig. 3). All considered variants were reported in common databases and genomic search engines such as VarSome [44], OMIM [45], ClinVar [46] or HGMD [47] and their consequence was assessed as known disease-causing variant, risk factor, variant of uncertain significance (VUS) or benign according to the ACMG guidelines for interpretation of sequence variants [48]. Five variants are predicted to be benign and are intronic or synonymous, three of them are classified as variants of uncertain significance and are missense or located in 3’UTR regions.

### Statistical analysis of variants frequency

For each of the 1046 variants detected, allele frequency was calculated. We calculated separately allele frequencies in the sAD and in the gCJD group. The latter was further divided according to the presence of the p.Glu200Lys or p.Val210Ile mutations. Each allele frequency was then compared to those reported into the GnomAd database [31] for the European (non-Finnish) population. Differences between observed and expected allele frequency were tested for statistical significance with Fisher’s Exact test and Benjamini–Hochberg multiple test correction. Table 2 summarizes the number of each type of variants per group and show the average number of variants per patient in the different classes (gene list reported in Additional file 1: Table S4).

**Table 2:** Summary of results of statistical analysis on each variant detected in our target sequencing panel

Disease group	Average number of SNV per patient	Unique SNV per disease group	Unique non-synonimous SNV per disease group		Unique SNV p < 0,05 per disease group
AD (46)	145.05	654	27		72
CJD (66)	134.87	768	11		33
E200K (26)	138.73	483	14		52
V210I (40)	135.73	645	27		75

Rows identify pathologic groups with their numerosity reported between brackets. The first column shows the average number of variants carried per patient in the different disease groups. The second column shows the overall number of different variants detected in each group in at least one patient. The third column indicates variants annotated as missense, splice variants or 3’or 5’ UTR in each disease group. The last column contains the number of variants with a p < 0.05 after Fisher’s exact test and Benjamini–Hochberg correction despite of their annotation

### Pathway analysis and protein–protein interaction network

**Fig. 4:**
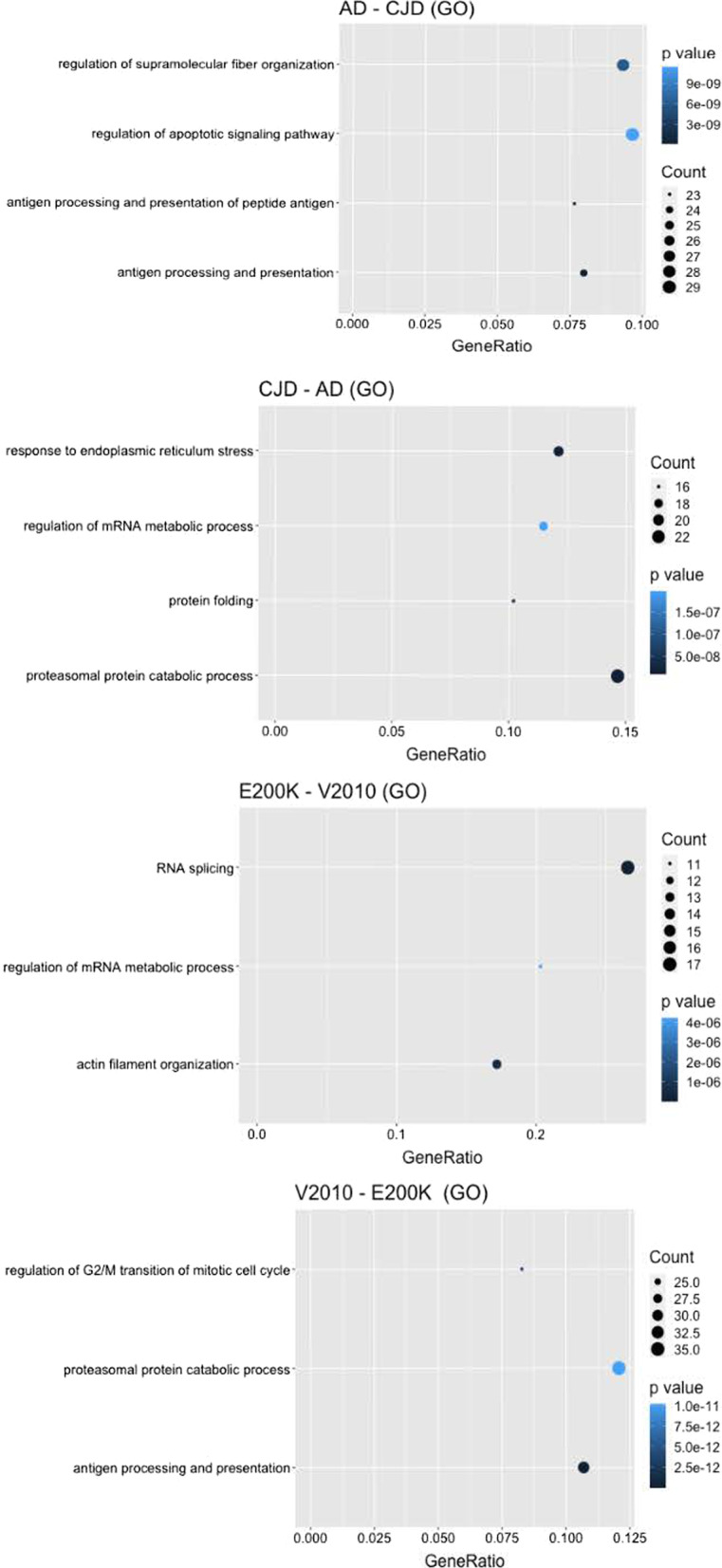
Result of functional enrichment analysis performed on genes harbouring variants with significantly altered allele frequency compared to European population reported in the GnomAd database. Results of pathway analysis are reported as significantly (p < 0.05) enriched pathways in the first group but not in the second of each coupled comparison. Since part of the affected pathways are shared among the considered conditions, results are reported as differences between comparisons of two groups. Complete results of the functional analysis with Gene Ontology and of the Protein–Protein Interaction networks are reported in Supplementary materials

To have functional insights of the consequences of the alterations in allele frequencies, genes harbouring at least one variant with p < 0.05 were used as input for pathway analysis with GO database (Fig. 4) and protein–protein interaction (PPI) network. Since part of the affected pathways are shared among the considered conditions, results are reported as differences between comparisons of two groups. Comparison of sAD vs gCJD in the PPI network shows a clear centrality of interactions of *APP, PSEN2* and *APOA1* in the AD but not in the CJD group (PPIn tables and figure in Additional file). Functional analysis of the same coupled comparison points out a significant (p < 0.05) enrichment in the sAD group (compared to gCJD) of the GO terms involving regulation of the apoptotic signaling pathway, supramolecular fiber organization, antigen processing and presentation of exogenous peptide antigen. Interestingly, in the CJD group we found and enrichment of GO terms involving the ER responses to stress, protein folding, regulation of mRNA maturation and splicing and in the regulation of catabolic processes. We then investigated whether functional differences within the gCJD group could provide further understanding of the different penetrance of the two mutations. In the coupled comparison between V210I and E200K, we found that only in the V210I group there is an enrichment of GO terms referring to proteasome mediated catabolic processes and antigen processing and presentation. In the PPIn results for the comparison V210IvsE200K, *APOA1* and *MAPT* together with *DCTN1* represented hubs of the network, highlighting a similarity between the enriched modules in the networks of the lowly penetrant p*.*Val210Ile mutation and the one of sAD, with numerous interactions and shared nearest neighbours involved in the enriched pathways. In the E200K group compared to the V210I we found a significantly altered regulation of mRNA and splicing, reflected in the PPI network by the abundant presence of members of the family of heterogeneous nuclear ribonucleoproteins (*hnRNPs* gene family) as nearest-neighbours of the input genes, in addition to the alteration of actin filament organization.

## Discussion

In this work, we addressed the challenge of exploring the complete genetic information carried by target sequencing data to acquire deeper insights in the genetic contributors of complex diseases. For this purpose, we selected a population of one-hundred and twelve patients affected by two neurodegenerative diseases: sporadic (sAD) and genetic Creutzfeldt–Jakob Disease (gCJD), either due to a highly (p.Glu200Lys) or a lowly (p.Val210Ile) penetrant mutation. As supported by previous research about genetic modifiers in this field, it is possible that other factors reinforce its pathogenic role in carriers of the *PRNP* p.Val210Ile who indeed develop CJD. In sAD, no specific causative mutations are present, nevertheless GWAS have revealed many loci of common genetic variation that confer risk for developing the disease and evidence supports a polygenic contribution to disease risk from common genetic variants [13, 49–51]. Our approach is based on a binary transformation of each detected variant. On the resulting matrix we applied statistical, supervised and unsupervised machine learning methods and network analysis as an unbiased approach to discover recurrent patterns and possible genetic modifiers among the genes included in the target sequencing. In this work we refer to patients’ groups as V210I, E200K and sAD groups, whilst specific protein coding variants are reported with the aminoacidic shift nomenclature and non-coding variants with nucleotide shift nomenclature.

### Unsupervised machine learning methods

Unsupervised methods identify as the main sources of variation in the dataset the haplotypes in *MAPT* and *NOTCH3* genes. Specifically, a set of haplotype-defining SNPs in *NOTCH3* do not follow the Hardy–Weinberg equilibrium law in our complete cohort suggesting a role of *NOTCH3* in the analysed neurodegenerative diseases. This role seems to be more stressed in the sAD group, both in the increasing number of variants with altered allele frequency (22 SNPs) and in the p-values when tested for HW equilibrium (p = 0.085). This result is in line with the functional role of *MAPT* haplotypes in most neurodegenerative diseases [43, 52–57] and with the role that *NOTCH3* in AD has been addressed by multiple previous reports [53, 58–61]. Thus, these results support the validity of our approach. Nevertheless, it must be considered that these results are dependent on the selected genes of the target sequencing and on the amount of SNVs present in those genes.

### Supervised machine learning methods

Supervised methods correctly classified our samples according to the two causative mutations responsible for the genetic forms of CJD. When applied to the data deprived of the two causative mutations, decision trees classified the phenotypic groups with 78% accuracy according to eight variants (Fig. 3 and Table 1). To our knowledge, none of the variants have been previously linked to the considered conditions. Four of these variants were not previously reported in the GnomAD database in the genes *APP,* c.*1A > C (rs748508166), *GRN,* c.1179 + 100A > T, *DCTN1,* p.Lys519Glu, *PRKAR1B,* c.595 + 369 T > C (rs1342588350), two of them are rare variants in the European population, *APP* p.Phe435 = (rs148180403, MAF = 0.001), *DCTN1* p.Ala816 = (rs1130484, MAF = 0.007) and two are common benign variants in *CHCHD10* (c.261 + 99A > G) and *GSN* (c.666 + 53 T > C). Six variants were carried only by patients with either sAD or gCJD (gini = 0.0): of these, five rare or VUS variants were found only in a subset of sAD patients (the two variants in *APP,* the two in *DCTN1* and the one in *PRKAR1B*), while the benign variant in *CHCHD10* was found only in gCJD. Despite the lack of statistical power of the study, it is reasonable that at least some of these variants could play a role as a contributor to the disease risk, given that both CJD mutations are not completely penetrant [62] and the polygenic nature of sAD [21]. Decision trees have been recently proposed as a suitable method in clinical applications and precision medicine for interpreting the role of genetic variants in complex diseases [63]. Our results reinforce the importance of this supervised method to improve understanding of the role of the numerous variants of uncertain significance and as a promising path towards precision medicine applications. In addition, our results indicate that decision trees can provide accurate classification on high-dimensional genomic data.

### Statistical and functional analysis

Statistical analysis performed on detected allele frequencies compared to those reported in the gnomAD database for the European non-Finnish population identified 33 to 75 variants with significantly altered allele frequency in each studied group. Each group showed a unique set of significant variants in the tested genes. Coherently with the hypothesis of a polygenic contribution in sAD, we found a higher number in sAD patients compared to gCJD both in the average number of variants per patient, with on average 145 variants carried by patients with sAD compared to the 134.87 in the genetic CJD group, and in the overall number of SNV with a significantly altered allele frequency (72 in sAD, 33 in gCJD, see Table 2 and Additional file 1: Table S4). These genes were used as input to perform functional analysis with Gene Ontology (Fig. 4) and PPI network comparing groups of interest, namely the two disease groups gCJD-sAD and the V210I-E200K CJD. In the first comparison, the PPI network identified important hubs only in the sAD group in correspondence of crucial genes in AD such as *APP*, *PSEN2* and *APOA1* despite the AD cohort did not bear any causative mutation. These results, together with the GO terms “regulation of the apoptotic signaling pathway”, “supramolecular fiber organization”, “antigen processing” and “presentation of exogenous peptide antigen” enriched in the functional analysis, are in line with previous reports about the polygenic nature of sAD and with its impaired pathways [50, 64–66]. With the same approach, in the CJD group we found an enrichment of pathways reported in previous functional studies as altered in this pathology, such as endoplasmic reticulum impairment, protein folding and regulation of mRNA maturation [67–70]. These results prove the validity of this approach to handle and valorise the great amount of information contained in target sequencing data and to acquire new insights about new putative risk variants. Within the CJD group, we observed differences in the lists of genes carrying altered allele frequencies, that were reflected in the functional analysis. The V210I-E200K coupled comparison showed differences between the genetic background of the same pathology triggered by different mutations. In the E200K-CJD compared to the V210I-CJD we found an altered regulation of mRNA and splicing, reflected in the PPI network by the abundant presence of members of the family of heterogeneous nuclear ribonucleoproteins (*hnRNPs* gene family) as nearest-neighbours of the input genes, in addition to the alteration of actin filament organization. Interestingly, a similarity between the affected pathways in V210I-CJD and sAD emerged: these two groups show a high number of variants with altered allele frequencies, 75 and 72, that lead in both cases in significant alterations in pathways involving proteasome-mediated catabolic processes, antigen processing and presentation, and PPI networks sharing various hubs, such as *APOA1* and *DCTN1*. These results are in line with several previous works that claim a complex genetic background in which the reinforcing role of several variants acting together increases the risk of developing a disease both in sporadic and in genetic forms [21, 59, 67, 71]. Here, functional pathway enrichment analysis and protein–protein interaction network showed a significant alteration in genes involved in immunity, catabolic processes, RNA splicing and cytoskeletal structure maintenance. These pathways are known to be altered in both AD and CJD [66, 68, 72–74] and in other neurodegenerative conditions, suggesting a contribution of those variants in exacerbating the pathologic alteration in those pathways.

## Conclusions

This work proposes an innovative approach towards the analysis of targeted NGS data, based on a binary transformation of the detected variants, on which an unbiased analysis is performed through statistical, machine learning and network analysis. Our results show that this method is a valuable workflow to explore recurrent genetic patters in homogenous phenotypic groups and increase our understanding of complex diseases. This approach can also be used to acquire new hints to identify specific SNV that could act as modifiers or risk factors in the studied condition. Specifically, we showed in our cohort how unsupervised methods can identify functionally relevant sources of variation in the data and that supervised classifiers can recognize clinical phenotypes with high accuracy based on the mutational profile of patients, thus representing a possible starting point for advanced diagnostic tools. Statistical, functional and network analysis provided functional insights that showed reliability in identifying both important known molecular features of the considered diseases and providing new insights on putative new genetic contributors. To conclude, we propose this workflow for an advanced analysis of target sequencing data in complex diseases.

## Supplementary Information

**Additional file 1.** Supplementary material and results: Identification of recurrent genetic patterns from targeted sequencing panels with advanced data science: a case-study on sporadic and genetic neurodegenerative diseases.

## Data Availability

The VCF files supporting the conclusions of this article are available in the European Variation Archive-EMBL-EBI (https://www.ebi.ac.uk/eva/), Project: PRJEB47822, Analyses: ERZ3614541 (https://www.ebi.ac.uk/ena/data/view/PRJEB47822).

## Abbreviations

NGS: Next generation sequencing
ML: Machine learning
SNV: Single nucleotide variant
SNP: Single nucleotide polymorphism
SAD: Sporadic Alzheimer Disease
GCJD: Genetic Creutzfeldt–Jakob disease
MAF: Minor allele frequency
PPIn: Protein–protein interaction network
PCA: Principal component analysis
