# Supplementary material for: Identification of recurrent genetic patterns from targeted sequencing panels with advanced data science: a case-study on sporadic and genetic neurodegenerative diseases
Source: BMC Med Genomics. 2022 Feb 10;15:26. doi: 10.1186/s12920-022-01173-4 (PMC8830183; doi:10.1186/s12920-022-01173-4)
Supplement: Supplementary file 1 — Additional file 1. Supplementary material and results: Identification of recurrent genetic patterns from targeted sequencing panels with advanced data science: a case-study on sporadic and genetic neurodegenerative diseases. [file 12920_2022_1173_MOESM1_ESM.docx]

**Supplementary materials and results: Identification of recurrent genetic patterns from targeted sequencing panels with advanced data science: a case-study on sporadic and genetic neurodegenerative diseases**

| **GENE SYMBOL** | **GENE NAME** | **ROLE** |
| --- | --- | --- |
| PSEN2 | Presenilin 2 | AD causing |
| PSEN1 | Presenilin 1 | AD causing |
| APP | Amyloid Beta Precursor Protein | AD causing |
| TYROBP | TYRO protein tyrosine kinase-binding protein | AD risk factor |
| TREM2 | triggering receptor expressed on myeloid cells 2 | AD risk factor |
| APOA1 | apolipoprotein 1 | AD risk factor |
| TARDBP | TAR DNA-binding protein | involved in FTD/ALS |
| CHMP2B | charged multivesticular body protein 2B | involved in FTD/ALS |
| VCP | valosin-containing protein | involved in FTD/ALS |
| FUS | FUS RNA-binding protein | involved in FTD/ALS |
| GRN | Granulin precursor | involved in FTD/ALS |
| MAPT | microtubule-associated protein tau | involved in FTD/ALS |
| OPTN | optineurin | involved in FTD/ALS |
| TBK1 | TANK-binding kinase 1 | involved in FTD/ALS |
| SQSTM1 | sesquestosome 1 | involved in FTD/ALS |
| HRNRPA2B | heterogeneous nuclear ribonucleoprotein A2/B1 | involved in FTD/ALS |
| PRKAR1B | protein kinase cAMP-dependent type I regulatory subunit beta | involved in FTD/ALS |
| CHCHD10 | coiled-coil-helix-coiled-coilhelix domain containing 10 | involved in FTD/ALS |
| SIGMAR1 | sigma nonopioid intracellular receptor 1 | involved in FTD/ALS |
| SNCA | synuclein alpha | involved in LBD |
| SERPINI1 | serpin family I member 1 | involved in other ND |
| CSF1R | colony stimulating factor 1 receptor | involved in other ND |
| ITM2B | integral membrane protein 2B | involved in other ND |
| DCTN1 | dynactin subunit 1 | involved in other ND |
| UBQLN2 | Ubiquilin 2 | involved in other ND |
| NOTCH3 | neurogenic locus notch homolog protein 3 | involved in vascular dementia |
| GSN | gelsolin | involved in vascular dementia |
| TTR | transthyretin | involved in vascular dementia |
| PRNP | Prion protein | Prion diseases causing |

**Table S1**: Genes covered in the target sequencing panel.


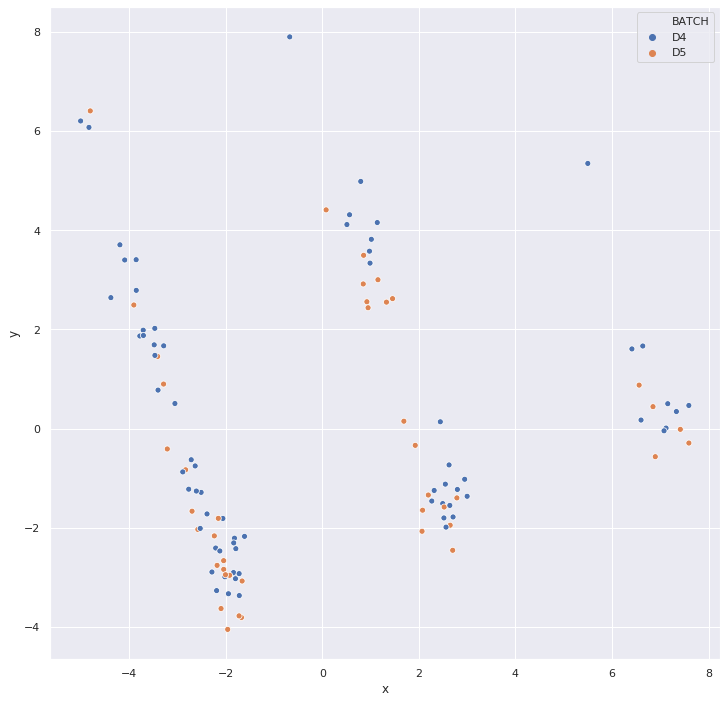

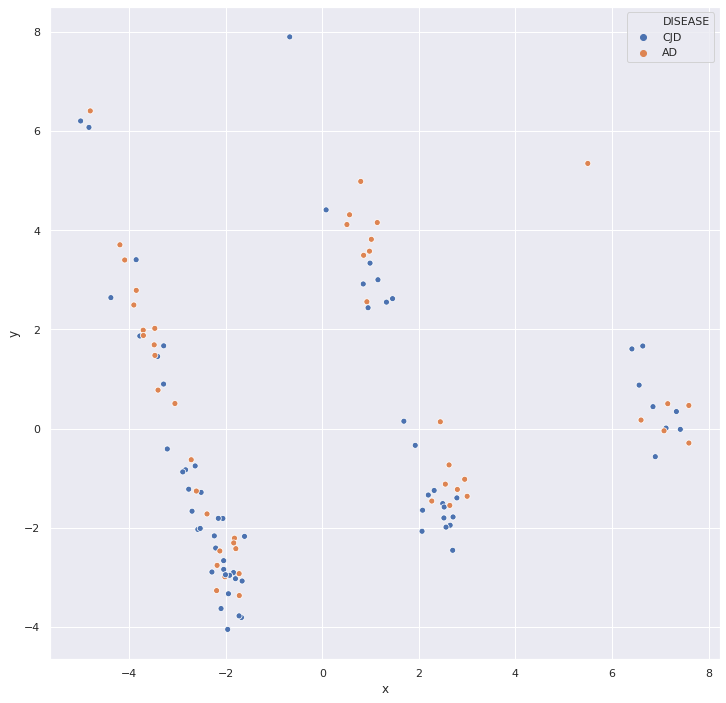


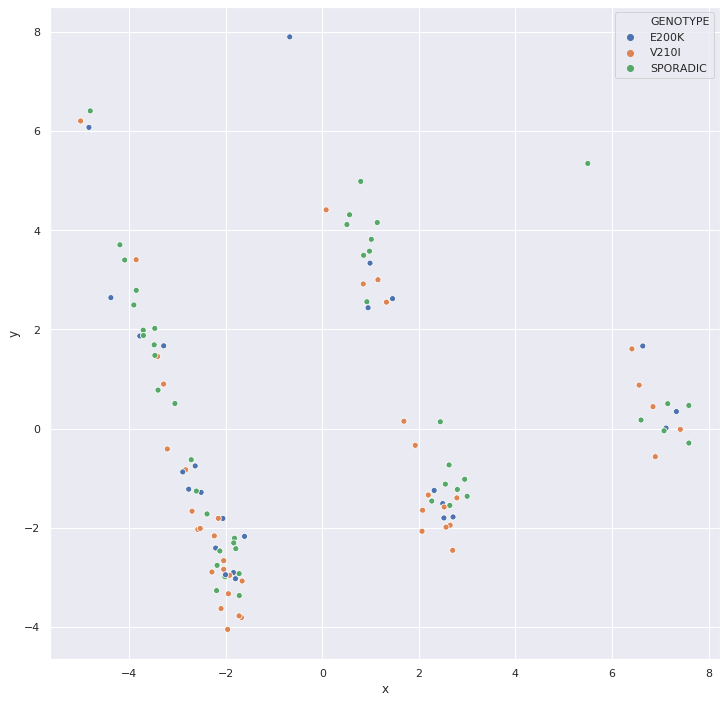


**Figure S1**: Superimposition of labels of batches, diseases and genotypes on the PCA plot.


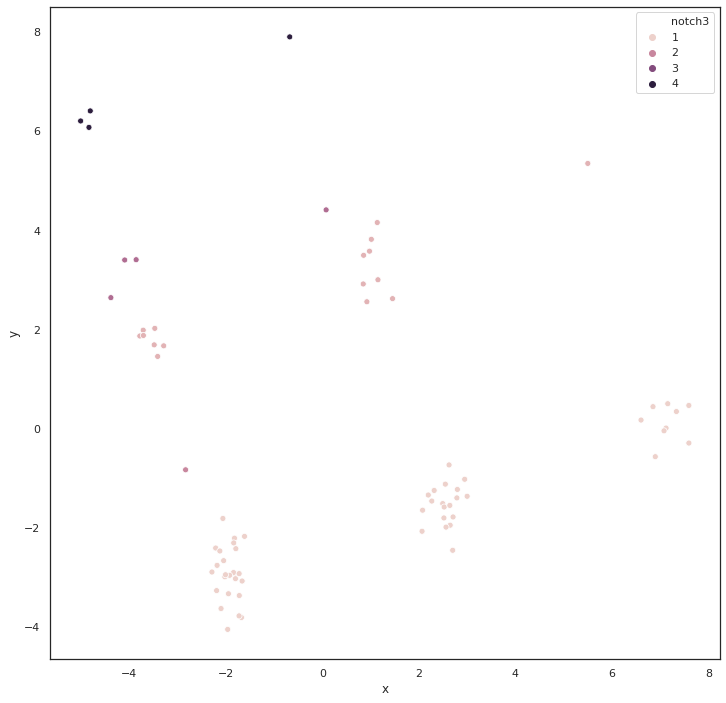


**Figure S2**: superimposition of labels of available NOTCH3 haplogroups on the PCA plot.

| variant | x | y |
| --- | --- | --- |
| chr17-43971785-A-G | 0.185702 | 0.067174 |
| chr17-43972025-TC-T | 0.191209 | 0.039996 |
| chr17-43971937-C-A | 0.194484 | 0.05385 |
| chr17-44073973-T-C | 0.197249 | 0.065004 |
| chr17-44039691-A-G | 0.197249 | 0.065004 |
| chr17-44067400-T-C | 0.197249 | 0.065004 |
| chr17-44067508-A-G | 0.197249 | 0.065004 |
| chr17-44068924-G-A | 0.197249 | 0.065004 |
| chr17-44091724-G-A | 0.197249 | 0.065004 |
| chr17-44049329-C-T | 0.197249 | 0.065004 |
| chr17-44061023-G-A | 0.197249 | 0.065004 |
| chr17-44071294-T-C | 0.197249 | 0.065004 |
| chr17-44067546-T-C | 0.197249 | 0.065004 |
| chr17-43972142-G-A | 0.197249 | 0.065004 |
| chr17-44061278-C-T | 0.197249 | 0.065004 |
| chr17-44073739-G-A | 0.197249 | 0.065004 |
| chr17-44051846-A-G | 0.197249 | 0.065004 |
| chr17-44061036-T-C | 0.197249 | 0.065004 |
| chr17-44101563-T-C | 0.197249 | 0.065004 |
| chr17-44073889-A-G | 0.197249 | 0.065004 |

**Table S2**: top 20 contributors to the first principal component

| **PC** | **Feature** |
| --- | --- |
| PC1 | chr17-43972142-G-A |
| PC2 | chr19-15289613-A-T |
| PC3 | chr1-227069737-C-T |
| PC4 | chr5-149441016-AC-A |
| PC5 | chr19-15302844-T-C |

**Table S3**: main contributors to the first five principal components (30% variance explained)

| **AD** | **CJD** | **E200K** | **V210I** |
| --- | --- | --- | --- |
| *OPTN* | *OPTN* | *OPTN* | *OPTN* |
| *APOA1* | *FUS* | *PSEN1* | *APOA1* |
| *PSEN2* | *MAPT* | *CCNF* | *ITM2B* |
| *FUS* | *PRNP* | *FUS* | *CCNF* |
| *MAPT* | *CHCHD10* | *TTR* | *FUS* |
| *NOTCH3* | *GSN* | *PRNP* | *MAPT* |
| *APP* |  | *DCTN1* | *NOTCH3* |
| *DCTN1* |  | *SERPINI1* | *PRNP* |
| *SERPINI1* |  | *SQSTM1* | *APP* |
| *CSF1R* |  | *GSN* | *DCTN1* |
| *PRKAR1B* |  |  | *CSF1R* |
| *GSN* |  |  | *TREM2* |
| *UBQLN2* |  |  | *PRKAR1B* |
|  |  |  | *GSN* |
|  |  |  | *VCP* |
|  |  |  | *UBQLN2* |

**Table S4: List of genes harbouring at least one variant with a significantly altered allele frequency in the different classes.** As reported in supplementary materials, in the CJD groups *PRNP* variants with significantly increased allele frequency were causative mutations and SNV at the codon 129, known modifier of prion diseases.
